# Antibody blockade of Dectin-2 suppresses house dust mite-induced Th2 cytokine production in dendritic cell- and monocyte-depleted peripheral blood mononuclear cell co-cultures from asthma patients

**DOI:** 10.1186/s12929-019-0598-6

**Published:** 2019-12-20

**Authors:** Ming-Han Chen, Ming-Ting Huang, Wen-Kuang Yu, Shinn-Shing Lee, Jia-Horng Wang, Ting-Jen R. Cheng, Michael R. Bowman, Shie-Liang Hsieh

**Affiliations:** 1 Division of Allergy, Immunology & Rheumatology, Department of Medicine, Taipei Veterans General Hospital, Taipei, Taiwan; 2 Department of Medicine, National Yang-Ming University, Taipei, Taiwan; 3 Genomics Research Center, Academia Sinica, Taipei, Taiwan; 4 Department of Chest Medicine, Taipei Veterans General Hospital, Taipei, Taiwan; 5 Section of Allergy, Immunology, and Rheumatology, Department of Medicine, Cheng Hsin Rehabilitation Medical Center, Taipei, Taiwan; 6 Critical Care, Far Eastern Memorial Hospital, Taipei, Taiwan; 7 Inflammation and Immunology Research Unit, Pfizer Inc, Cambridge, MA USA; 8 Present address: Immunology and Inflammation Therapeutic Area, Sanofi, Cambridge, MA USA; 9 Institute of Clinical Medicine, National Yang-Ming University, Taipei, Taiwan; 10 Department of Medical Research, Taipei Veterans General Hospital, Taipei, Taiwan; 11 Institute for Cancer Biology and Drug Discovery, Taipei Medical University, 128 Academia Road, Section 2, Nankang, Taipei, 115 Taiwan

**Keywords:** House dust mite, *Dermatophagoides pteronyssinus*, Dectin-2, Asthma

## Abstract

**Background:**

Dectin-2, which is a C-type lectin, interacts with the house dust mite (HDM) *Dermatophagoides pteronyssinus* allergen. This study aimed to investigate whether Dectin-2 blockade by antagonistic monoclonal antibodies (MoAbs) attenuates HDM-induced allergic responses.

**Methods:**

Two anti-Dectin-2 MoAbs were generated and validated for specific binding to Dectin-2 Fc fusion protein (Dectin-2.Fc) and inhibition of Dectin-2.Fc/HDM interaction. Patients with asthma exhibiting high titers of anti-*D. pteronyssinus* IgE were enrolled. Peripheral blood mononuclear cells with depleted CD14^+^ monocytes were obtained from these patients and co-cultured with autologous monocyte-derived conventional dendritic cells in the presence of *D. pteronyssinus* or its group 2 allergens (Der p 2). Interleukin (IL)-5 and IL-13 levels in the culture supernatants were determined using ELISA in the presence or absence of anti-Dectin-2 MoAbs.

**Results:**

Two MoAbs, 6A4G7 and 17A1D10, showed specific binding to recombinant Dectin-2.Fc and inhibited HDM binding to Dectin-2.Fc. Both anti-Dectin-2 MoAbs inhibited IL-5 and IL-13 production in co-cultures with Der p 2 stimulation in a dose-dependent manner. 6A4G7 and 17A1D10 (3 μg/mL) significantly inhibited Der p 2-induced (3 μg/mL) IL-5 production by 69.7 and 86.4% and IL-13 production by 84.0 and 81.4%, respectively. Moreover, this inhibitory effect of the two MoAbs remained significant in the presence of *D. pteronyssinus*.

**Conclusions:**

Anti-Dectin-2 MoAbs significantly inhibited HDM-induced allergic responses in vitro and therefore have the potential to become therapeutic agents in mite-induced allergic diseases.

## Introduction

Asthma is a chronic airway disease characterized by inflammation, bronchial hyperresponsiveness, and airway remodeling. In the epithelial environment stage, the lung epithelium contacts with inhaled allergens, acting as inflammatory promoters capable of helping dendritic cells (DCs) to skew T-cell differentiation toward a T-helper 2 (Th2) response [1–3]. The airway epithelial cells mediate airway immune response through the production of cytokines and chemokines, which are key contributors in the chemotaxis of neutrophils, eosinophils, basophils, mast cells, DCs, and T- and B-cells. Allergen-specific immunoglobulin (Ig) E antibodies, DCs, and Th2 cells have been thought to contribute to the pathophysiology of allergic reactions in the Th2 polarization stage [4, 5]. In particular, DCs promote Th2 responses by producing proinflammatory cytokines and upregulating the expression of co-stimulatory molecules following exposure to the house dust mite (HDM) *Dermatophagoides pteronyssinus* antigen [6–8]. Activated Th2 cells produce interleukin (IL)-5 and IL-13, which play important roles in the tissue damage stage. IL-5 is a critical mediator of eosinophil activation to promote bronchial inflammation and asthma symptoms, and IL-13 is involved in bronchial hyperreactivity and airway remodeling, such as mucus metaplasia and subepithelial fibrosis [9–13]. A growing body of evidence suggests that these two Th2 cytokines are potential therapeutic targets for allergic asthma [14].

Innate immune cells, such as DCs, are activated by foreign antigens via pattern recognition receptors, including Toll-like receptors and C-type lectin receptors (CLRs) [15, 16]. Dectin-2, a member of the Syk-coupled CLR group, is expressed in human monocytes and recognizes various fungal pathogens [17–19]. Dectin-2 activation induces inflammatory cytokine and chemokine production via the Syk-protein kinase C-δ (PKCδ)–CARD9 pathway [20–22].

Recently, Dectin-2 was further shown to interact with HDM allergen extracts and contributed to Th2 immunity following HDM activation [23, 24]. Moreover, Dectin-2 recognized extracts from the HDM species *D. pteronyssinus* and elicited Th2 responses through cysteinyl leukotrienes in mice [24]. In addition, Dectin-2 promoted HDM-induced Th2 differentiation but worsened allergic airway inflammation in a mouse model [25]. Dectin-2 regulated *D. farinae*-induced production of chemokine, including CCL4 and CCL8, in monocyte-derived DCs in an animal model of asthma, indicating that Dectin-2 is involved in both allergic sensitization and challenge phases [26]. Importantly, Dectin-2-positive infiltrating cells were detected in bronchial biopsies from patients with asthma [27]. Collectively, these results suggested that Dectin-2 plays critical roles in the pathogenesis of allergic diseases. Therefore, identification of the antagonistic Dectin-2 ligand and production of antagonistic MoAbs to block the ligand–Dectin-2 interaction would aid in the exploration of its mechanism in allergic diseases. Therefore, in this study, we generated anti-Dectin-2 MoAbs by immunizing mice and investigated their inhibitory effects on HDM-induced Th2 cytokine production in DC- and monocyte-depleted peripheral blood mononuclear cell (PBMC) co-cultures from patients with asthma.

## Materials and methods

### Generation of MoAbs against human Dectin-2

Human full-length Decin-2 was cloned from PBMC complementary DNA (cDNA) by polymerase chain reaction (PCR). The PCR primers used to amplify cDNA fragments encoding human Dectin-2 are as follows: sense, 5′ –AAGCTTGGATGATGCAAGAGCAGCAACCTC- 3′, and anti-sense, 5′ –GGATCCTCATAGGTAAATCTTATTCATCTCAC-3′. The amplified fragments were sequenced and subcloned in-frame into pTagRFP. The extracellular domain of Dectin-2 was fused with the fc portion of human IgG1 (Dectin-2.Fc), as described previously [28]. Anti-Dectin-2 MoAbs were selected via ELISA-based differential screening, and only those recognizing recombinant Dectin-2.Fc (100 ng of Dectin-2.Fc per well) but not human IgG1 were regarded as positive clones. In addition to ELISA, flow cytometry was used to confirm the binding ability and specificity of the selected MoAbs to Dectin-2. HEK 293 T (human embryonic kidney cells transformed with large T antigen) cells were cultured in complete DMEM (10% fetal calf serum (FCS)). Human Dectin-2 full length construct was transiently transfected into HEK 293 T cells using Lipofectamine 2000 (Invitrogen, Carlsbad, CA, USA) as suggested by manufacturer’s protocol. Human promyelocytic leukemia cells (HL-60) were cultured in RPMI-1640 supplemented with 10% FCS. Human PBMCs were isolated from healthy donors by standard density gradient centrifugation with Ficoll-Paque. To get primate peripheral blood leukocytes (PBLs), 2 ml fresh monkey *(Macaca cyclopis)* whole blood were lysed with RBC lysis buffer (0.826% NH_4_Cl, 0.1% KHCO_3_ and 1 mM EDTA) and incubating for 5 min at room temperature. After centrifugation, supernatants were removed and cell pellets were washed with PBS once before resuspended in FACS buffer for cell staining. For preparation of mouse bone marrow-derived DCs (BMDCs), BM cells were isolated from femurs and tibias and cultured in RPMI-1640 medium supplemented with 10% FCS and 20 ng/ml of mouse GM-CSF (R&D Systems, Minneapolis, MN, USA) for 7 days. On day 7, suspended cells were harvested and used as BMDCs. HEK 293 T cells overexpressing full-length human Dectin-2, HL-60 cells, human PBMCs, primate PBL cells, and mouse BMDCs were stained with anti-Dectin-2 MoAbs (1 μg/10^6^ cells) at 4 °C for 30 min. After washing with PBS, cells were incubated with FITC-conjugated anti-mouse IgG at 4 °C and analyzed by flow cytometry (FACSCantoII, Becton Dickinson, Mountain View, CA, USA). Next, the interaction between anti-Dectin-2 MoAbs and Dectin-2.Fc was measured based on surface plasmon resonance using a biosensor Biacore T200 instrument (GE Healthcare, Biacore, Freiburg, Germany). Briefly, Dectin-2.Fc was immobilized onto a CM5 BIAcore sensor chip, and purified anti-Dectin-2 MoAbs 6A4G7 and 17A1D10 were injected into each sensor cell at a flow rate of 30 μL/min and association and dissociation times of 2 and 10 min, respectively. The MoAb 7652A3, which cannot bind to Dectin-2, was used as a control. Data were processed by zeroing the time and response before MoAb injection and by subtracting a reference channel injected with the running buffer alone.

### Inhibition of Dectin-2.Fc–HDM interaction by anti-Dectin-2 MoAbs

Anti-Dectin-2 MoAbs were tested for their ability to inhibit Dectin-2.Fc binding to a HDM *D. pteronyssinus*-coated plate. *D. pteronyssinus* extract (lot number 215941, 23.0 EU/mg endotoxin, Greer Laboratories, Inc., Lenoir, NC, USA), at a final concentration of 30 μg/mL in 100 mM sodium bicarbonate (pH 9.6), was incubated with polymyxin B (PMB) (Sigma Cheical Co., St. Louis, MO, USA), at a final concentration of 1 μg/mL of at 37 °C for 1 hh, to remove lipopolysaccharide (LPS) contamination and was coated overnight at 4 °C onto 96-well plates at 50 μL per well. After washing with Tris-buffered saline (TBS), the wells were blocked with 1% bovine serum albumin (Sigma Chemical Co.) in TBS for 1 hour at room temperature and then washed for at least three times with TBS. Anti-Dectin-2 MoAb or control IgG2a (Sigma Chemical Co.) was pre-incubated with 10 μg/mL Dectin-2.Fc for 20 min, followed its addition to non-coated or *D. pteronyssinus*-coated plates at various concentrations (3.75, 7.5, 15, and 30 μg/mL). Following incubation at room temperature for 2.5 h, wells were washed for three times with TBS. HRP-conjugated goat anti-mouse MoAb (1:5000 dilution; Catalog no. AP181P; Millipore, Burlington, MA, USA) was added for 30 min at room temperature, followed by washing with TBS before the addition of TMB; this mixture was incubated for 30 min at room temperature. A stop solution (2NH_2_SO_4_) was added, and absorbance was measured at 450 nm. Percent inhibition by anti-Dectin-2 MoAbs was calculated using the following formula: Inhibition (%) = [1 − OD (sample with anti-Dectin-2 MoAb) / OD (sample with IgG2a)] × 100. The results were expressed as mean percent inhibition of duplicate measurements.

### Patients and healthy controls

This study was approved by the institutional ethics committee of Taipei Veterans General Hospital (VGHTPE-IRB 2014–10-005CC) and Cheng Hsin Rehabilitation Medical Center (CHGH-IRB 373–102-20). Informed consent was obtained for all participants in this study. All patients with asthma presented a history of asthma, as confirmed by airway obstruction reversibility, and the healthy controls presented no history of or findings consistent with allergic diseases. Patients exhibiting high titers of anti-*D. pteronyssinus* IgE (> 0.5 kU/L) were enrolled. The Asthma Control Test (ACT) score, ranging from 5 to 25, was used to assess asthma severity [29].

### Monocyte isolation and DC differentiation

PBMCs were isolated using density gradient centrifugation with Ficoll-Paque™ plus (Amersham Pharmacia Biotech, Uppsala, Sweden). Subsequently, monocytes were purified using the VarioMACS technique with anti-CD14 microbeads (Miltenyi Biotec GmbH, Bergisch Gladbach, Germany) as per the manual’s instructions. Briefly, PBMCs were incubated with anti-CD14 magnetic beads at 4 °C for 30 min. After washing, the cell suspension was loaded into a column and placed in the magnetic field of a MACS separator (Miltenyi Biotec). CD14^−^ PBMCs were obtained as the flow-through fraction after binding to anti-CD14 microbeads. Immature DCs were generated by culturing CD14^+^ monocytes for 6 days in a complete RPMI 1640 medium supplemented with 10% FCS and 50 μM 2-mercaptoethanol (Life Technologies, Gaithersburg, MD, USA), containing 25 ng/mL human GM-CSF (Leucomax®, Schering-Plough, Kenilworth, NJ, USA) and 20 ng/mL human IL-4 (R&D Systems). Half of the volume of complete RPMI 1640 medium was added on day 3, and suspended immature DCs (PBMC–immature DCs) were harvested on day 6.

### Determination of Th2 cytokine production in DC and CD14^−^ PBMC co-culture

Monocyte-derived DCs (MoDCs) and thawed autologous CD14^−^ PBMCs were co-cultured at a ratio of 1:8 in 96-well flat-bottom plates on RPMI 1640 medium supplemented with 10% FCS. HDM *D. pteronyssinus* (Greer Laboratories, Inc.) and its group 2 allergens (Der p 2) (Indoor Biotechnologies, Inc., Charlottesville, VA, USA) were pre-incubated with PMB (Sigma Chemical Co.) at a final concentration of 1 μg/mL at 37 °C for 1 h. LPS-depleted *D. pteronyssinus* or Der p 2 was added to the co-cultures at a final concentration of 0, 1, or 3 μg/mL and incubated for 6 days. These cells were further stimulated with the same concentration of allergens for another 6 days, and IL-5 and IL-13 levels in the supernatants were measured using ELISA (R&D Systems). The experiments were performed in duplicate.

### Assay of anti-human Dectin-2 MoAb-mediated inhibition of HDM-induced Th2 cytokine production

Assessment of the antagonistic activity of anti-Dectin-2 MoAbs was based on an in vitro assay using *D. pteronyssinus* or recombinant Der p 2 to stimulate Th2 cytokine production in a DCs and CD14^−^ PBMC co-culture, as described previously [23]. Anti-Dectin-2 MoAbs were added at a final concentration of 0.3, 1, or 3 μg/mL; IgG2a at a final concentration of 3 μg/mL was used as an isotype control. After 6 days of co-culture, the supernatant was removed and cells were co-cultured with the same concentration of allergens and MoAbs for another 6 days. The supernatant was harvested, and IL-5 and IL-13 levels were measured using ELISA. Percent inhibition of cytokine release was calculated as follows: 100 × (cytokine level with IgG2a treatment − cytokine level with anti-Dectin-2 MoAb treatment) / (cytokine level with IgG2a treatment). The experiments were performed in duplicate.

### Statistical analysis

Data were analyzed using two-tailed Student’s t-test for continuous parametric variables and Mann–Whitney *U* test for nonparametric variables. Chi-squared test was used to compare percentages between groups. Spearman’s correlation was used to assess the association between two variables. Two-tailed *p* values were calculated, and *p* < 0.05 was considered significant. All statistical analyses were performed using SPSS (SPSS 15.0 for Windows, SPSS, Chicago, IL, USA).

## Results

### Selection of MoAbs that bound to Dectin-2

**Fig. 1:**
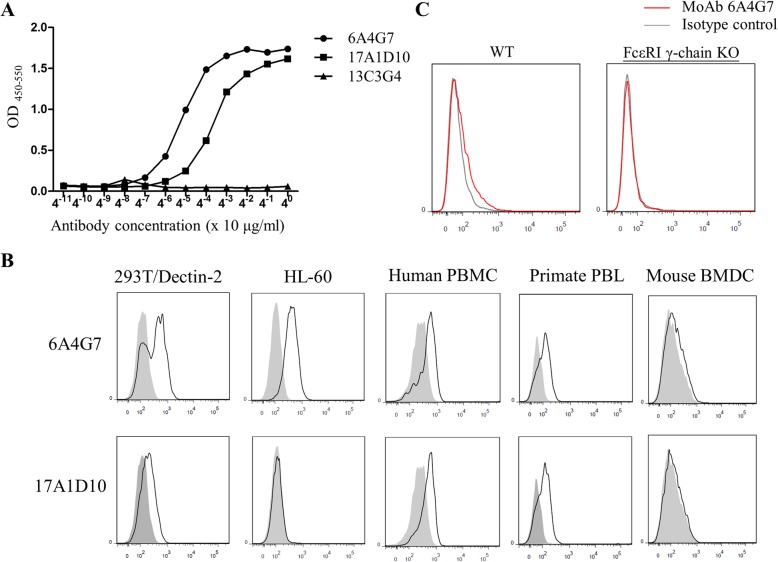
Binding of anti-Dectin-2 monoclonal antibodies to Dectin-2, (**a**) The binding curve of monoclonal antibodies (MoAbs) to recombinant Dectin-2.Fc, as measured by ELISA. Microtiter plates were coated with 1 μg/mL of Dectin-2.Fc. Thereafter, MoAbs were added to the wells at a series of concentrations. A secondary goat anti-mouse MoAbs-conjugated horseradish peroxidase (HSP) was added, and colorimetric detection was performed using tetramethylbenzidine (TMB) substrate. After adding the stop solution, absorbance was measured at 450 nm using a spectrophotometer. The MoAbs 6A4G7 and 17A1D10 bound to Dectin-2.Fc in a dose-dependent manner, whereas the control MoAb 13C3G4 did not. **b** HEK 293 T cells were transduced with pTagRFP/Dectin-2 or an empty vector. The binding of 6A4G7 MoAb (top) and 17A1D10 MoAb (bottom) to HEK 293 T cells transfected with Dectin-2 (open histogram) or empty vector (shaded histogram) was determined by flow cytometry. Data are representative of two independent experiments. **c** Anti-Dectin-2 MoAb 6A4G7 could bind to Dectin-2 on mouse bone marrow-derived DCs (BMDCs). BMDCs were cultured from C57BL/6 (wild-type, WT) and FcεRI γ-chain knockout (KO) mice, which does not express Dectin-2 on the cell surface. BMDCs were stained with anti-Dectin-2 MoAbs (1 μg/10^6^ cells) at 4 °C for 30 min. After washing with PBS, cells were incubated with fluorochrome-conjugated anti-mouse IgG at 4 °C for 30 min. After washing with PBS, cells were fixed with 1% paraformaldehyde. Binding was analyzed by flow cytometry

Both 6A4G7 and 17A1D10 successfully bound to Dectin-2, as demonstrated by ELISA and flow cytometry. Moreover, both MoAbs did not bind to the other C-type lectins or Syk-associated receptors. Both 6A4G7 and 17A1D10 bound to Dectin-2.Fc in a dose-dependent manner (Fig. 1a), whereas the control antibody 13C3G4 did not under the same conditions. The binding ability to 6A4G7 to Dectin-2.Fc was higher than that of 17A1D10. Flow cytometry confirmed that both anti-Dectin-2 MoAbs recognized human Dectin-2 (Fig. 1b) because they reacted with Dectin-2-transduced HEK 293 T cells but not with HEK 293 T cells transduced with an empty vector. Similarly, both 6A4G7 and 17A1D10 bound to human PBMC and primate PBLs. Although 6A4G7 bound to human HL-60 cells strongly, its binding to wild-type BMDC (C57BL/6) was much weak. 6A4G7 did not bind to the FcεRI γ-chain-deficient BDMCs (Fig. 1c), which did not express mouse Dectin-2 [30]. Therefore, we concluded that the 6A4G7 showed a mild cross-reaction to mouse Dectin-2.We also evaluated the binding activity of anti-Dectin-2 MoAbs to human MoDCs and T cells. As shown in Additional file 1: Figure S1A, we can detect Dectin-2 expression in MoDCs by 6A4G7 and 17A1D10. In addition, the results showed that anti-Dectin-2 MoAbs can’t bind to human CD3 + CD4+ or CD3 + CD8+ T cells from PBMCs (Additional file 1: Figure S1B).

### Kinetics of anti-Dectin-2 MoAb binding to Dectin-2.Fc

We further determined the binding affinity of anti-Dectin-2 MoAbs to Dectin-2.Fc by surface plasma resonance using a BIAcore biosensor. We found that 6A4G7 and 17A1D10 but not isotype control 7652A3 bound to immobilized Dectin-2.Fc with high affinity (3.9 × 10^− 9^ and 71 × 10^− 8^ M, respectively) (Table 1). Compared with the high-affinity MoAb 6A4G7, the low-affinity MoAb 17A1D10 bound to the Dectin-2.Fc with slower kinetic association (8.5 × 10^6^ M^− 1^·s^− 1^ vs. 4.4 × 10^4^ M^− 1^·s^− 1^) and dissociation rates (2.9 × 10^− 2^ s^− 1^ vs. 2.3 × 10^− 3^ s^− 1^). This observation demonstrated that the binding affinity of 6A4G7 to Dectin-2.Fc was higher than that of 17A1D10.

**Table 1:** Kinetic analysis of the binding of the monoclonal antibodies 6A4G7 and 17A1D10 to the Dectin-2.Fc fusion protein

Anti-Dectin-2 monoclonal antibody	*K*_a_ (association rate) (M^−1^·s^− 1^)	*K*_d_ (disassociation rate) (s^− 1^)	*K*_D_ (disassociation constant) (M)
6A4G7	8.5 (± 6.4) × 10^6^	2.9 (± 1.6) × 10^− 2^	3.9 (± 0.9) × 10^− 9^
17A1D10	4.4 (± 2.8) × 10^4^	2.3 (± 0.2) × 10^− 3^	7.1 (± 2.1) × 10^− 8^

Adherence of anti-Dectin-2 monoclonal antibodies to Dectin-2.Fc was measured in duplicate

### Inhibited of binding of recombinant Dectin-2.Fc to *D. pteronyssinus* by anti-Dectin-2 MoAbs

**Fig. 2:**
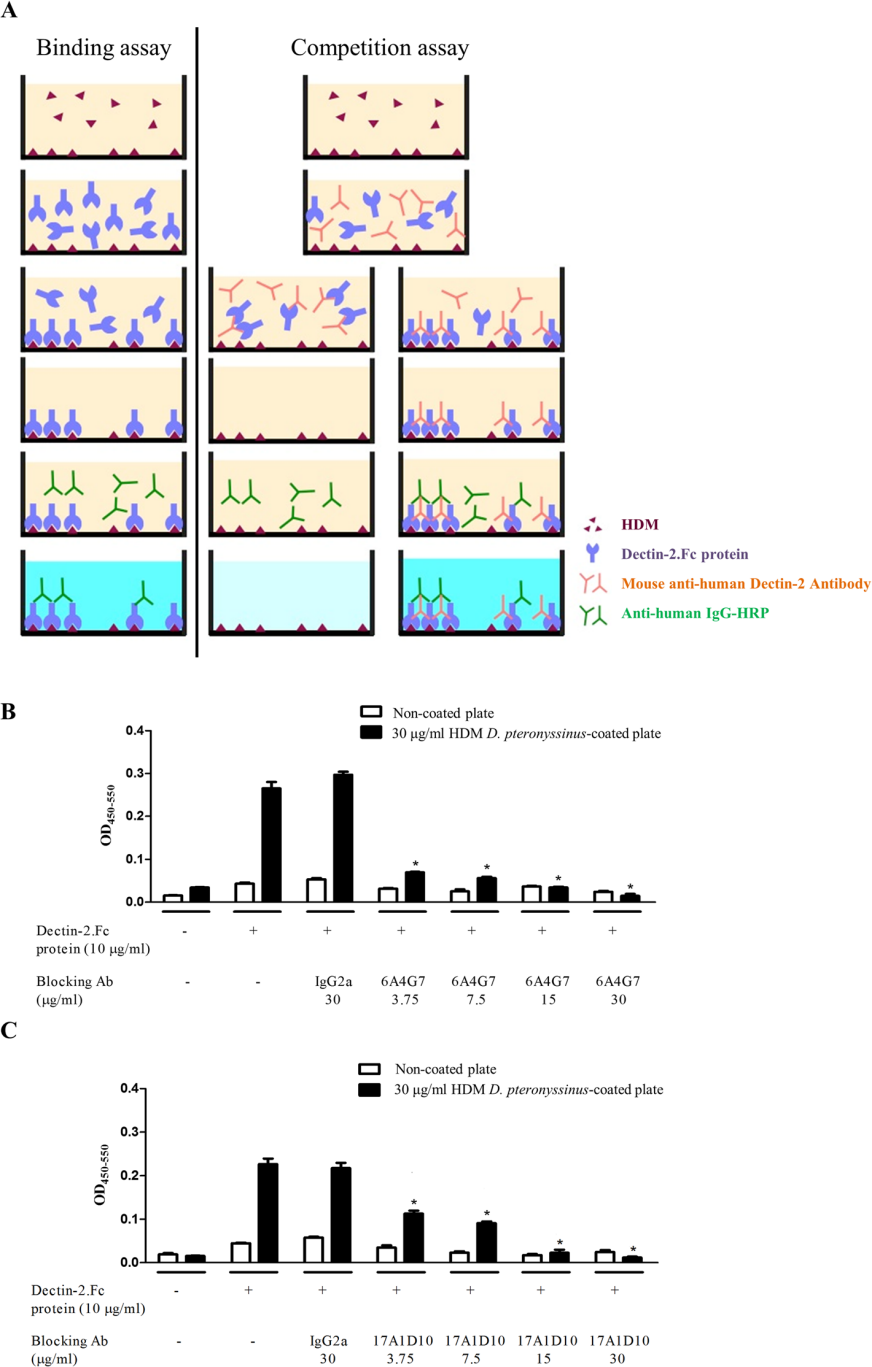
Competitive blocking assay for anti-Dectin-2 monoclonal antibodies in Dectin-2.Fc fusion protein to house dust mite, (**a**) The house dust mite *Dermatophagoides pteronyssinus* was coated and placed on an ELISA plate; then, the anti-Dectin-2 MoAbs 6A4G7 and 17A1D10 were added to compete the binding between *D. pteronyssinus* and Dectin-2.Fc. **b** and **c** Dectin-2.Fc fusion protein (10 μg/mL) was incubated on either the non-coated plates or the *D. pteronyssinus*-coated plates in the presence or absence of the anti-Dectin-2 MoAb 6A4G7 (**b**) or 17A1D10 (**c**) and IgG2a. HRP-conjugated goat anti-mouse MoAb was added, and absorbance at 450 nm was measured using a spectrophotometer. In non-coated plates (white column), binding of Dectin-2.Fc with any of the MoAbs was not observed. Conversely, Dectin-2.Fc was found to bind to *D. pteronyssinus*-coated plates (black column) in the presence and absence of IgG2a. Student’s t-test was used to compare MoAb treatment with the control IgG2a. * *p* < 0.05

The results of competition assay of inhibition of Dectin-2.Fc binding to *D. pteronyssinus* by anti-Dectin-2 MoAbs are depicted in Fig. 2a. Dectin-2.Fc did not bind to the non-coated plates regardless of the presence of anti-Dectin-2 MoAbs (Fig. 2b and c); however, it strongly bound to *D. pteronyssinus*-coated plates in the absence of anti-Dectin-2 MoAbs. The binding ability of Dectin-2.Fc to *D. pteronyssinus*-coated plates decreased in the presence of 6A4G7 (Fig. 2b) and 17A1D10 (Fig. 2c) in a dose-dependent manner. 6A4G7 inhibited Dectin-2.Fc binding to *D. pteronyssinus* at a half maximal inhibitory concentration (IC_50_) of 1.11 μg/mL, which was lower than that for 17A1D10 (IC_50_ = 4.04 μg/mL). The isotype control MoAb IgG2a did not exert any inhibitory effects of *D. pteronyssinus*–Dectin-2 binding. Both 6A4G7 and 17A1D10 exhibited dose-dependent inhibition of recombinant Dectin-2.Fc binding to *D. pteronyssinus*. These observations showed that 6A4G7 was more potent than 17A1D10 in inhibiting Dectin-2 binding to *D. pteronyssinus*.

### Clinical characteristics of patients with asthma and healthy controls

Demographic, laboratory, and clinical characteristics of 12 patients with asthma and 4 healthy controls enrolled are summarized in Table 2. Mean age was 50.1 years for patients with asthma and 54.3 years for healthy controls. Median duration of disease for patients with asthma was 20.0 years. Mean levels of total IgE and IgE specific to the HDM *D. pteronyssinus* antigen were 574.3 and 11.1 kU/L in patients with asthma and 15.5 and 0.09 kU/L in healthy controls, respectively. PEF results showed that the % predicted values of forced expiratory volume in 1 s (FEV_1_) and FEV_1_/forced vital capacity (FVC) were 81.1 and 69.7%, respectively. Mean ACT score was 18.3 (12–24) points.

**Table 2:** Demographic and clinical characteristics of patients with asthma and healthy controls

Determinants	Normal range	Patients with asthma	Healthy controls
Number		12	4
Age in years, mean (SD)		50.1 (11.4)	54.3 (8.8)
Sex, male, n (%)		7 (58.3)	2 (50.0)
Disease duration in years, medium (range)		20.0 (0.5–59)	–
Laboratory profiles			
Total eosinophil count (/μL), mean (SD)	<450.0	340.0 (236.6)	124.8 (42.6)
Total IgE (kU/L), mean (SD)	<200.0	574.3 (1126.1)	15.5 (16.5)
Specific anti-*D. pteronyssinus* IgE (kU/L), mean (SD)	<0.5	11.1 (11.1)	0.09 (0.08)
Pulmonary function test			
% of predicted FEV_1_, mean (SD)	>80.0	81.1 (14.9)	–
% of predicted FEV_1_/FVC, mean (SD)	>70.0	69.7 (9.5)	–
Anti-asthma drugs			
Bronchodilator, n (%)		12 (100.0)	–
Leukotriene antagonist, n (%)		2 (16.7)	–
Corticosteroid, n (%)		4 (33.3)	–
Asthma Control Test score, mean (range)^a^		18.3 (12–24)	–

Abbreviation: *FEV*_*1*_ Forced expiratory volume in 1 s, *FVC* Forced vital capacity, *SD* Standard deviation

^a^Asthma control test score ranges from 5 to 25, with higher scores reflecting greater asthma control

### Inhibition of HDM-induced Th2 cytokine production by anti-Dectin-2 MoAbs

**Fig. 3:**
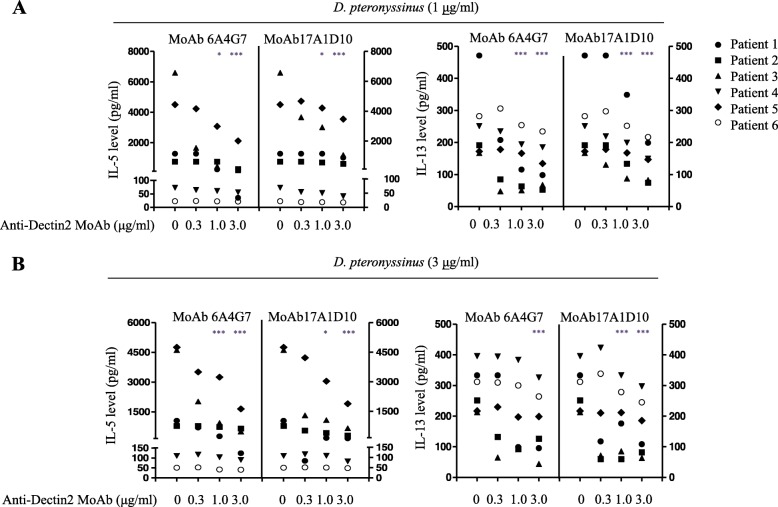
Anti-Dectin-2 monoclonal antibodies inhibited *Dermatophagoides pteronyssinus*-stimulated T-helper 2 cell cytokine production in monocyte-derived DC/CD14^−^ PBMC co-culture, PBMCs were obtained from six patients with asthma with high level of anti-D. pteronyssinus IgE. Monocyte-derived DCs (MoDCs) and CD14^−^ PBMCs were prepared as described in the Materials and Methods section. *D. pteronyssinus* was added to the co-cultures of MoDCs and CD14^−^ PBMC at a final concentration of 1 (**a**) or 3 (**b**) μg/mL. Anti-Dectin-2 monoclonal antibody (MoAb) 6A4G7 or 17A1D10 was added at a final concentration of 0.3, 1, or 3 μg/mL. IgG2a, at a final concentration of 3 μg/mL, was used as an isotype control. After 6 days of co-culture, the supernatant was removed, and the cells were co-cultured with the same concentration of *D. pteronyssinus* and anti-Dectin-2 MoAbs for another 6 days. The supernatant was harvested, and IL-5 and IL-13 levels were measured by ELISA. Mann–Whitney *U* test was used to compare the percent inhibition of cytokine release between anti-Dectin-2 MoAb and IgG2a. * *p* < 0.05; ** *p* < 0.01; *** *p* < 0.005

**Fig. 4:**
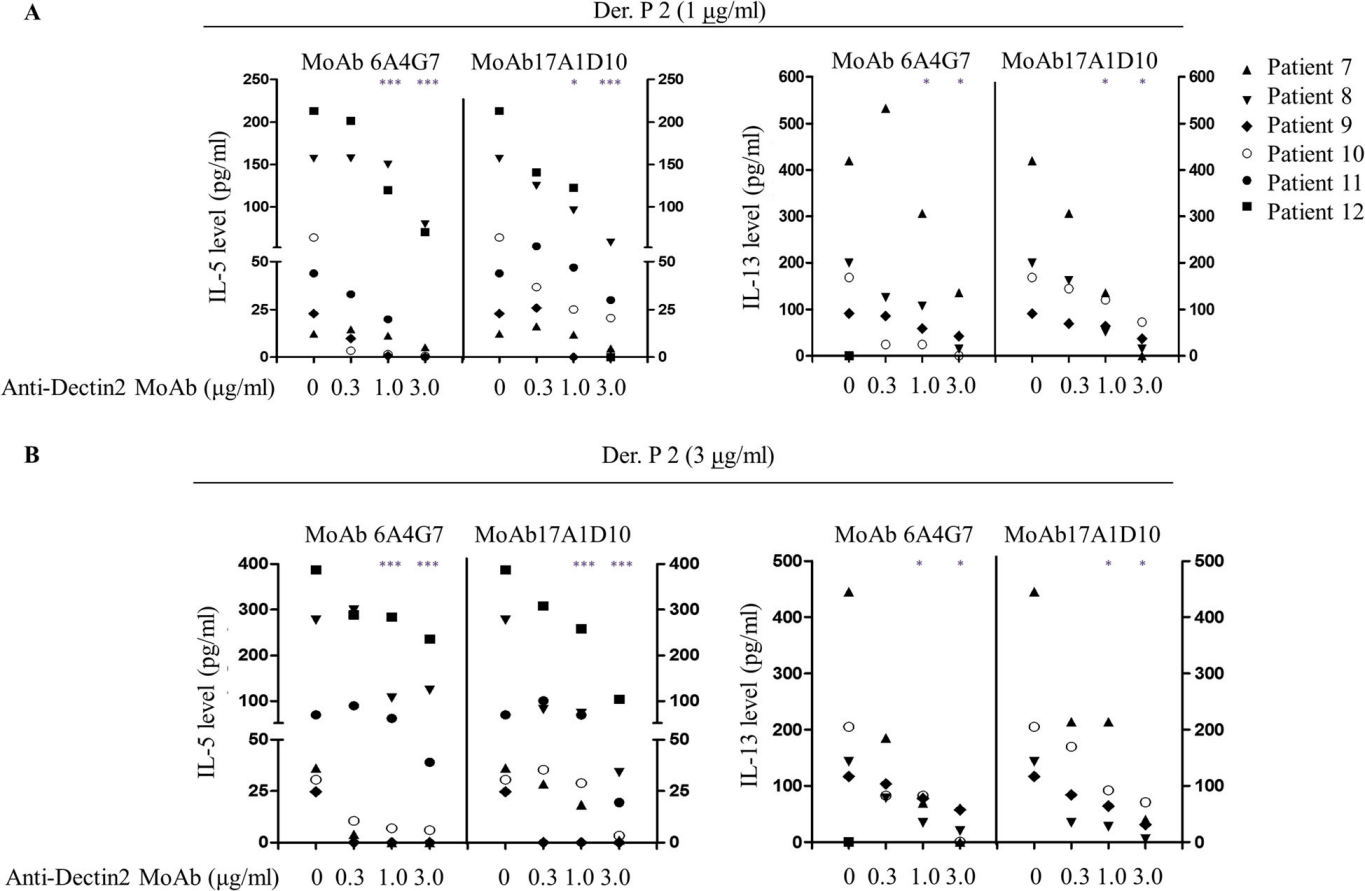
Anti-Dectin-2 monoclonal antibodies inhibited Der p 2-stimulated T-helper 2 cell cytokine production in monocyte-derived DC/CD14^−^ PBMC co-culture, PBMCs were obtained from six patients with asthma with high level of anti-D. pteronyssinus IgE. Monocyte-derived DCs (MoDCs) and CD14^−^ PBMCs were prepared as described in the Materials and Methods section. Der p 2 was added to the co-cultures of MoDCs and CD14^−^ PBMC at a final concentration of 1 (**a**) or 3 (**b**) μg/mL. Anti-Dectin-2 monoclonal antibody (MoAb) 6A4G7 and 17A1D10 were added at a final concentration of 0.3, 1, or 3 μg/mL. IgG2a, at a final concentration of 3 μg/mL, was used as an isotype control. After second stimulation, the supernatant was harvested and IL-5 and IL-13 levels were measured by ELISA. Mann–Whitney *U* test was used to compare the percent inhibition of cytokine release between anti-Dectin-2 MoAb and IgG2a. * *p* < 0.05; ** *p* < 0.01; *** *p* < 0.005

To examine whether Dectin-2 blockade affected Th2 cytokine production in *D. pteronyssinus-* or Der p 2-stimulated DCs, CD14^−^ PBMCs obtained from patients with asthma were incubated with MoDCs in the presence of *D. pteronyssinus* or Der p 2. In Der p 2 stimulation, substantial IL-5 and IL-13 levels were detected in most patients with asthma but not in healthy controls; IL-5 production was detectable in all patents with asthma, and IL-13 was detectable in 10 of the 12 patients with asthma. Next, serial dilutions of the anti-Dectin-2 MoAbs were added to the co-culture to determine their inhibitory effects on *D. pteronyssinus-* or Der p 2-stimulated Th2 cytokine production. 6A4G7 showed dose-dependent inhibition of *D. pteronyssinus-* or Der p 2-induced IL-5 production in MoDC/CD14^−^ PBMC co-cultures was observed in all patients with asthma (Figs. 3 and 4, respectively). In co-cultures of six patients, compared with IgG2a, 6A4G7 (0.3, 1.0, and 3.0 μg/mL) inhibited IL-5 production by 17.7, 35.9, and 49.3%, respectively, following *D. pteronyssinus* (3 μg/mL) stimulation (Table 3); even higher inhibition rates of 46.6, 62.6, and 69.7%, respectively, were observed when following stimulation by Der p 2 (3 μg/mL). Similarly, 6A4G7 dose-dependently inhibited IL-13 production following stimulation with *D. pteronyssinus* or Der p 2.

**Table 3:** Inhibition of IL-5 and IL-13 production in monocyte-derived dendritic cells/CD14^−^ peripheral blood mononuclear cell co-culture in HDM stimulation by anti-Dectin 2 monoclonal antibody in patients with asthma

Anti-Dectin-2 MoAb	6A4G7			17A1D10		
Concentration (μg/mL)	0.3	1.0	3.0	0.3	1.0	3.0
*Dermatophagoides pteronyssinus* (1 μg/mL)						
Inhibition of IL-5 (%)	14.7	37.3^*^	56.7^***^	12.5	18.2^*^	34.2^***^
Inhibition of IL-13 (%)	29.6	41.4^***^	46.1^***^	4.3	22.9^***^	41.3^***^
*Dermatophagoides pteronyssinus* (3 μg/mL)						
Inhibition of IL-5 (%)	17.7	35.9^***^	49.3^***^	32.0	39.6	53.3^***^
Inhibition of IL-13 (%)	18.7	34.3^***^	40.2^***^	32.5	35.4^***^	44.3^***^
Der p 2 (1 μg/mL)						
Inhibition of IL-5 (%)	30.3	51.1^***^	79.0^***^	16.1	41.1^*^	71.0^***^
Inhibition of IL-13 (%)	32.1	48.6^*^	78.4^*^	20.9^*^	50.0^*^	77.3^*^
Der p 2 (3 μg/mL)						
Inhibition of IL-5 (%)	46.6	62.6^***^	69.7^***^	35.4	43.7^***^	86.4^***^
Inhibition of IL-13 (%)	43.7^*^	63.4^*^	84.0^*^	43.3^*^	58.2^*^	81.4^*^

Abbreviation: *MoAb* Monoclonal antibody, *HDM* House dust mite, *IL* Interleukin, *Der p 2 D. pteronyssinus* or its group 2 allergens

The percent inhibition of cytokine release was calculated as follows: 100 × (cytokine level with IgG2a treatment − cytokine level with anti-Dectin-2 MoAb treatment) / (cytokine level with IgG2a treatment)

Mann–Whitney *U* test was used to compare the percent inhibition of cytokine release between anti-Dectin-2 MoAb and IgG2a

* *p* < 0.05; ** *p* < 0.01; *** *p* < 0.005

Similar to 6A4G7, 17A1D10 inhibited *D. pteronyssinus* or Der p 2-stimulated IL-5 and IL-13 production in the MoDC/CD14^−^ PBMC co-cultures of patients with asthma (Figs. 3 and 4, respectively). The dose-dependent inhibitory effects of 17A1D10 in MoDC/CD14^−^ PBMC co-cultures are summarized in Table 3.

Together, these observations suggest that the anti-Dectin-2 MoAbs 6A4G7 and 17A1D10 specifically inhibited Th2 cytokine production in the patients with asthma.

## Discussion

In this study, we successfully generated two MoAbs, 6A4G7 and 17A1D10, which can specifically bind to Dectin-2 and competitively block the binding of the recombinant chimeric Dectin-2.Fc to HDM. In addition, these two MoAbs dose-dependently inhibited allergen-induced IL-5 and IL-13 production in patients exhibiting high titers of anti-*D. pteronyssinus* IgE. In support of the report that IL-5 and IL-13 antibodies, such as mepolizumab and lebrikizumab, respectively, demonstrated certain clinical efficacy in a subset of patients with asthma [31, 32], our findings suggested that anti-Dectin-2 MoAbs can be potential therapeutics for mite-mediated allergic diseases via blocking IL-5 and IL-13 production.

Asthma has a complex pathogenesis, and DCs play an important role in it. Lung DCs capture and present antigens to Th2 cells, thereby initiating allergic Th2 cell responses to inhaled allergens. Moreover, DCs produce chemokines, which recruit T-cells, eosinophils, and basophils into the lungs as well as co-stimulatory molecules that aid the activation of antigen-specific T-cells [33]. In previous studies, depletion of DCs from the airway of mice was shown to prevent asthma development, as revealed by the inhibition of eosinophilic inflammation and Th2 cytokine production [34–36]. Dectin-2, a pattern recognition receptor for fungi, is selectively expressed on DCs and macrophages [17, 18]. Recent data revealed that Dectin-2 also recognizes HDM and induces cysteinyl leukotrienes production by DCs to elicit a Th2 response in mouse models [23, 24]. In this study, we demonstrated that Dectin-2 MoAbs can inhibit the binding of purified Dectin-2 protein to *D. pteronyssinus*-coated plates and HDM antigen-induced production of Th2 cytokines, suggesting these antibodies can block the binding of HDM antigen to Dectin-2 on human DCs.

IL-5 and IL-13, which are secreted mainly by Th2 cells and group 2 innate lymphoid cells, play important roles in the pathophysiology of asthma. Increased IL-5 and IL-13 expression has been found in allergen-challenged bronchoalveolar lavage fluid cells of patients with asthma [37, 38]. Moreover, IL-5 has been implicated in enhanced eosinophilic accumulation, activation, and survival as well as aggravated bronchial inflammation and asthma symptoms [9, 10]. Meanwhile, IL-13 reportedly promoted the differentiation of naïve T-cells to Th2 cells, activating isotype switching toward IgE production in allergen-specific B-cells, contributed to mucous metaplasia, and enhanced the remodeling of airway walls in patients with asthma [5, 11–13]. MoAbs against IL-5 reduce asthma exacerbations and thus show the potential to serve as therapeutic agents in a selected population of patients with asthma [31, 39]. Improvement in FEV_1_ has been observed in patients with asthma with a high serum periostin levels or detectable sputum IL-13 levels following the administration of MoAbs against IL-13 [32, 40]. In patients with asthma exhibiting high anti-*D. pteronyssinus* IgE levels, anti-Dectin-2 MoAbs substantially reduced HDM allergen-induced Th2 cytokine production by an average of more than 60%. The combined inhibition on IL-5 and IL-13 by these anti-Dectin-2 MoAbs was consistent with the proposed mechanism that Dectin-2 is an upstream receptor critical for HDM-induced Th2 response. Therefore, the inhibition of Dectin-2-mediated inflammation may provide a robust approach for treating allergic diseases by simultaneously reducing IL-5 and IL-13 production.

Previous studies found that corticosteroids suppress IL-5 and IL-13 production. Dexamethasone downregulated antigen-induced IL-5 and IL-13 expression in human PBMCs [41]. Similarly, glucocorticoids inhibited IL-13 transcription directly and indirectly [42, 43], as well as suppressed IL-5 transcription [44]. However, there is a lack of evidence regarding the influence of asthma medication, including corticosteroids, on IL-5 and IL-13 production in a co-culture system. There were no significant differences in IL-5 and IL-13 production in our co-culture system under stimulation by *D. pteronyssinus* or Der p 2 between patients treated with and without corticosteroids or leukotriene antagonists (all *p* > 0.05). These results may attributable to the limited sample size in this study. Further research is thus warranted to compare IL-5 and IL-13 production in the same population before and after asthma treatment.

Asthma pathogenesis is complex and involves immune cells and inflammatory cascades. Several therapeutic antibodies that bind and neutralize their molecular targets have been developed, including anti-IgE (omalizumab and ligelizumab), anti-IL-5 (mepolizumab and reslizumab), anti-IL-13 (tralokinumab and, lebrikizumab), anti-CCL11 (bertilimumab), and anti-IL-4 receptor α-chain (dupilumab) [45]. However, there is no single cytokine or cell responsible for complete asthma pathogenesis. Therefore, a challenge of MoAb therapy in asthma treatment is that the medications may benefit only a small proportion of patients. In addition to HDM, Dectin-2 recognizes *Aspergillus fumigatus* hyphae and triggers the Syk-CARD9 signaling pathway in plasmacytoid DCs to activate adaptive immune responses [19, 20]. Moreover, Dectin-2 plays an important role in host defense against *Candida albicans* [46]*.* Therefore, a possible side effect of anti-Dectin-2 MoAbs includes the attenuation host immune responses induced by fungal infection.

IL-4, another Th2 cytokine, mediates the development of allergic inflammation, including the induction of the IgE isotype switch in human B-cells and vascular cell adhesion molecule-1 (VCAM-1) on endothelial cells, promotion of eosinophil transmigration across cytokine-activated endothelial cells, and differentiation of Th2 cells [47]. IL-4 and IL-13 as well as the IL-4 receptor complexes that they bind to play key roles in the pathogenesis of allergic diseases [48]. Indeed, dupilumab, a humanized monoclonal antibody against IL-4 receptor α-chain, has been proven to treat severe atopic dermatitis [49] and may be beneficial against asthma [50]. Barrett et al. reported that *D. farinae*-elicited Th2 cytokine generation (including IL-4, IL-5, and IL-13) is dependent on Dectin-2 [24]. However, IL-4 levels in the supernatant were extremely low in our co-culture system. Further studies is thus warranted to test the effects of Dectin-2 blockade on HDM-induced IL-4 generation.

This study has several limitations. First, the number of patients included was relatively small; hence, further studies with a larger sample size are required to confirm our findings. Second, the institutional ethics committees in this study impose a blood draw limit at a single time point; thus, other cytokines or chemokines were not assayed.

## Conclusion

In this study, we demonstrated that anti-Dectin-2 MoAbs considerably inhibit HDM-induced IL-5 and IL-13 production in DC- and monocyte-depleted PBMC co-cultures of patients with asthma. Our findings provide an alternative therapeutic target for mite-induced allergic diseases.

## Supplementary information

**Additional file 1.** Anti-Dectin-2 MoAbs 6A4G7 and 17A1D10 can bind to monocyte-derived dendritic cells but not T cells. (A) CD14+ monocyte-derived dendritic cells isolated from healthy donors were prepared as described in the Materials and Methods section. Cells were stained with anti-Dectin-2 monoclonal antibodies (MoAbs) 6A4G7, 17A1D10 (1 μg/10^6^ cells) or an isotype control IgG2a and analyzed by flow cytometry. (B) Peripheral blood mononuclear cells isolated from healthy subjects were stained with MoAbs binding to CD3, CD4, CD8, anti-Dectin-2 MoAb 6A4G7 or 17A1D10 (1 μg/10^6^ cells) or isotype control IgG2a at 4 °C for 30 minutes. After washing with PBS, the cells were fixed with 1% paraformaldehyde. Binding was analyzed by flow cytometry. Cells were gated on CD3+CD4+ and CD3+CD8+ T cells. The experiments were performed in duplicate.

## Data Availability

The data that support the findings of this study are available from the corresponding author upon reasonable request.

## Abbreviations

ACT score: Asthma Control Test score
BMDCs: Bone marrow-derived dendritic cells
CLEC5A: C-type lectin member 5A
CLRs: C-type lectin receptors
*D. pteronyssinus*: *Dermatophagoides pteronyssinus*
DCs: dendritic cells
Der p 2: *Dermatophagoides pteronyssinus* group 2 allergens
FEV_1_: Forced expiratory volume in 1 s
FVC: Forced vital capacity
HDM: House dust mite
IL-13: Interleukin-13
IL-5: Interleukin-5
LPS: Lipopolysaccharide
MoAbs: Monoclonal antibodies
PBMCs: Peripheral blood mononuclear cells
PKCδ: Protein kinase C-δ
PMB: Polymyxin B
TBS: Tris-buffered saline
TH2 cells: T-helper 2 cells
