# Supplementary figures and images for: Antibody blockade of Dectin-2 suppresses house dust mite-induced Th2 cytokine production in dendritic cell- and monocyte-depleted peripheral blood mononuclear cell co-cultures from asthma patients
Source: J Biomed Sci. 2019 Dec 20;26:97. doi: 10.1186/s12929-019-0598-6 (PMC6925444; doi:10.1186/s12929-019-0598-6)

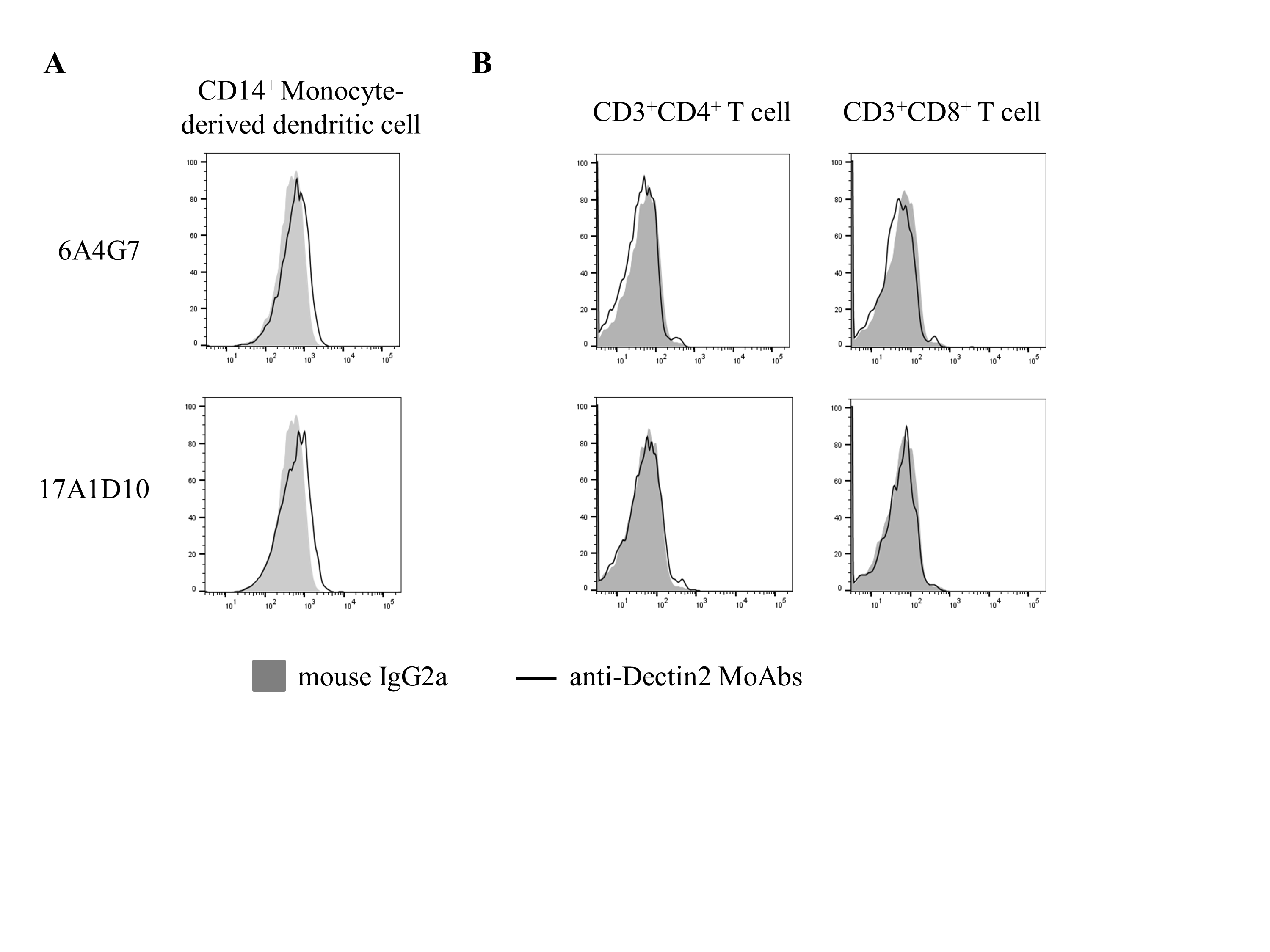

Supplement: Supplementary file 1 — Additional file 1. Anti-Dectin-2 MoAbs 6A4G7 and 17A1D10 can bind to monocyte-derived dendritic cells but not T cells. (A) CD14+ monocyte-derived dendritic cells isolated from healthy donors were prepared as described in the Materials and Methods section. Cells were stained with anti-Dectin-2 monoclonal antibodies (MoAbs) 6A4G7, 17A1D10 (1 μg/106 cells) or an isotype control IgG2a and analyzed by flow cytometry. (B) Peripheral blood mononuclear cells isolated from healthy subjects were stained with MoAbs binding to CD3, CD4, CD8, anti-Dectin-2 MoAb 6A4G7 or 17A1D10 (1 μg/106 cells) or isotype control IgG2a at 4 °C for 30 minutes. After washing with PBS, the cells were fixed with 1% paraformaldehyde. Binding was analyzed by flow cytometry. Cells were gated on CD3+CD4+ and CD3+CD8+ T cells. The experiments were performed in duplicate. [file 12929_2019_598_MOESM1_ESM.tif]
